# Drawing therapy based on embodied cognition theory on emotional expression and social behavior in students with autism: a mixed-methods study

**DOI:** 10.3389/fpsyg.2025.1664699

**Published:** 2025-10-23

**Authors:** Guanghui Li, Daren Wei, Yalong Xing, Yan Li, Wu Song

**Affiliations:** ^1^Faculty of Innovation and Design, City University of Macau, Macau, China; ^2^School of Art and Design, Jimei University, Xiamen, China; ^3^School of Fine Arts, Nanjing Normal University, Nanjing, China

**Keywords:** embodied cognition, autism spectrum disorder (ASD), drawing-based intervention, mixed-methods design, self-concept reconstruction, cultural symbols

## Abstract

**Objective:**

Autism spectrum disorder (ASD) is often characterized by deficits in emotional expression and social functioning. Existing interventions tend to emphasize behavioral correction, often overlooking the role of bodily movement in cognitive reconstruction and neglecting the emotional-metaphorical function of cultural symbols that may limit therapeutic effectiveness.

**Methods:**

This mixed-method study randomly assigned 60 ASD students aged 6–19 into an intervention group (*n* = 30) and a control group (*n* = 30), which received a 9-week Embodied Cognition-Based Drawing Therapy (EC-DT), or a control group (*n* = 30) that continued routine training. Assessment tools included psychiatric diagnostic instruments, art-based drawing evaluations, and self-report scales (TSCS, GQOL-74). To complement the quantitative results, qualitative data were collected through semi-structured interviews and analysis of participants’ drawings, enabling a case-based evaluation of the intervention’s effectiveness.

**Results:**

Quantitative analyses revealed that the intervention group showed significantly greater improvements than the control group in self-concept (ΔTSCS = 29.37, *p* < 0.001), social functioning (ΔGAS = 15.6, *p* = 0.003), and quality of life (ΔGQOL-74 = 21.3, *p* < 0.001). Qualitative findings identified a “body–media–emotion” pathway, illustrating how participants regulated emotions through tactile engagement (e.g., “feeling emotions flow through the fingertips while drawing circles”) and embedded cultural elements (e.g., using red to symbolize warmth) to enhance emotional resonance and social connectivity.

**Conclusion:**

The EC-DT model significantly improves emotional expression, social behavior, and self-concept among autistic students through multisensory integration and culturally embedded embodied experiences. These findings support the development of localized, culturally responsive intervention frameworks. Further longitudinal research is needed to confirm the durability of these therapeutic effects.

## 1 Introduction

Art therapy, initially established by Margaret Naumburg in the mid-20th century, has emerged as a non-verbal therapeutic modality particularly suitable for autism spectrum disorder (ASD) intervention. Individuals with ASD typically exhibit deficits in verbal communication and social interaction, often accompanied by embodied cognitive impairments such as repetitive behaviors and sensorimotor dysfunctions (American Psychiatric Association, 2013). Through creative expression, art therapy circumvents language barriers and fosters emotional release and social adaptation (Bosgraaf et al., 2020; Czamanski-Cohen and Weihs, 2023).

Recent developments in embodied cognition theory have shed light on the cognitive mechanisms underlying the integration of artistic creation and bodily movement. Embodied cognition posits that cognition arises from the dynamic interplay between the body and its environment (Cornejo et al., 2017), aligning closely with the sensorimotor challenges observed in ASD (Abrahamson, 2014). Nevertheless, mainstream intervention approaches—such as Applied Behavior Analysis (ABA)—have largely prioritized behavioral modification, often overlooking the cognitive reconstructive potential of bodily engagement. In terms of technological integration, countries in Europe and North America have begun incorporating human-computer interaction (HCI) technologies into art therapy (Hirsch et al., 2024). However, reliance on pharmacological treatments remains a significant clinical concern due to associated side effects (Wang et al., 2025). In contrast, art therapy within China’s special education landscape is still nascent, facing two primary limitations: first, the predominance of Western theoretical paradigms; second, the insufficient exploration of localized cultural elements—such as ink wash techniques and traditional symbols—within embodied cognitive frameworks (Cui and Wang, 2022).

Notably, the emotional and symbolic functions of cultural motifs (e.g., red symbolizing “auspiciousness,” or circular forms indicating “reunion”) have not been fully utilized, constraining the cross-cultural adaptability of existing interventions. To address these gaps, the present study sets out to achieve three objectives: (1) construct a drawing-based intervention model grounded in embodied cognition (EC-DT) and quantitatively evaluate its effects on core ASD symptoms; (2) uncover the interactive mechanisms between bodily movement and cultural symbolism; and (3) promote culturally contextualized practices by integrating traditional art elements to enhance social behavior and cultural identity in autistic students.

By embedding symbolic meanings from traditional Chinese art—such as brushwork, pattern metaphors, and emotional color associations—into intervention design, this study provides a distinct alternative to Western art therapy practices. For instance, variations in the weight, speed, and angle of brushstrokes, along with the layered and diffused effects of ink wash, highlight atmosphere and fluidity rather than strict representation. This emphasis on “spirit over form” (写意, *xie yi*) allows students with autism greater flexibility and freedom in emotional expression. Moreover, the intuitiveness and expressiveness of brushstrokes and ink flow can foster meditative and restorative experiences, offering a culturally grounded complement with cross-cultural relevance to mainstream Western approaches. Looking ahead, this rich artistic tradition could be integrated with digital tools (e.g., VR technology) and localized cultural symbolism, pointing to a promising direction for innovative and context-sensitive autism interventions.

## 2 Literature review

### 2.1 Embodied cognition theory

The word “embodiment” originates from the field of psychology, which means that cognitive processes not only rely on the brain, but are also directly affected by physical attributes and sensorimotor systems (Criscuolo et al., 2022). The theory of embodied cognition emphasizes that cognition is the result of the interaction between the body and the environment through perception and action (Farina, 2021). This view breaks through the “disembodiment” hypothesis of traditional cognitive science and provides a new perspective for autism intervention. The social impairment of autistic students is highly correlated with sensorimotor disorders (such as difficulty in motor coordination and tactile hypersensitivity) (Cornejo et al., 2017), and the theory of embodied cognition focuses on the cognitive reconstruction mechanism of such body-environment interaction defects, becoming an important theoretical fulcrum for art therapy.

### 2.2 Evolution and limitations of cognitive theories of autism

Traditional cognitive theories of autism primarily include Theory of Mind (ToM), Weak Central Coherence (WCC), and Executive Function (EF). ToM suggests that individuals with autism have difficulty understanding the mental states of others (Baron-Cohen et al., 1985). WCC proposes that their cognitive style is characterized by an excessive focus on details at the expense of global processing (Frith and Happé, 1994). EF theories emphasize impairments in planning, cognitive flexibility, and self-regulation (Hofmann et al., 2012). While these frameworks offer valuable insights into social interaction difficulties in autism—such as challenges in emotion recognition and atypical face processing strategies (Ji et al., 2025; Pandey and Bhushan, 2025), they inadequately address the role of bodily movement in cognitive development.

For instance, the ToM model struggles to explain how drawing activities may enhance empathic ability in autistic students (Kao et al., 2020). In contrast, embodied cognition theory fills this explanatory gap by emphasizing the “perception–action loop,” a mechanism through which sensorimotor engagement directly supports emotional and cognitive processing.

### 2.3 Integration of painting therapy theory

Painting therapy emerged in the early 20th century, with Freud and Jung emphasizing its function in releasing unconscious conflicts through visual expression (Kapitan, 2018). One of the earliest standardized assessments was the human figure drawing test developed by Koppitz (1966), which attempted to use painting for psychological evaluation. However, its reliability and validity—particularly in individuals with autism—remain controversial, especially regarding its limited predictive power for emotion recognition. Subsequent research improved test protocols using regression analysis, confirming that painting can serve as an effective tool for assessing emotional stress (Koppitz, 1966). In parallel, Chinese researchers explored case-based localized applications, contributing to culturally relevant practices (Chu et al., 2018).

Recent developments have incorporated embodied cognition theory into painting therapy, revealing a dual-intervention mechanism: motor actions drive cognitive integration, while group-based artistic activities foster social coordination. For instance, hand movements during painting activate the mirror neuron system and facilitate emotional expression (Rizzolatti and Craighero, 2004). Similarly, collective painting tasks involving synchronized movements (e.g., rhythmic brushwork) stimulate sensorimotor brain regions and enhance interpersonal coordination (Naeem et al., 2012).

However, many current intervention models overlook the moderating role of cultural symbols. While Western studies often emphasize abstract line and color expression, Chinese autistic students may communicate emotions through traditional ink painting techniques. Enabled by painting tools, the flowing medium and elegant style of ink painting contain profound cultural metaphors (Joschko et al., 2022). These differences highlight the need for localized theoretical frameworks that take into account cultural aesthetics and symbolic systems in therapeutic design.

### 2.4 Research gaps and innovative directions

While embodied cognition offers a novel paradigm for drawing-based interventions, several critical challenges remain. First, existing standardized assessment tools, such as the Koppitz human figure drawing test, lack sensitivity in capturing the emotional expressions of individuals with autism. These tools often rely on abstract symbolic systems that fail to reflect the atypical, sensory-dominated expressive behaviors common among students with autism. In terms of symbolic meaning, the color red symbolizes warmth and auspiciousness in Chinese culture, while in Western clinical contexts, it often connotes danger or warning (Chen and Chi, 2022). Likewise, Chinese “cloud patterns” and “reciprocating patterns” have unique philosophical meanings, representing “eternal cycles” and symbolizing harmony and peace (Yu-Ming et al., 2023). In contrast, Western interventions often employ abstract forms like jagged lines or zigzags, which lack resonance within Eastern cultural frameworks.

Empirical studies further underscore this divergence. Research has found that when Chinese autistic students were asked to draw the theme of “family,” a significant proportion spontaneously employed circular compositions to symbolize reunion. In contrast, comparable studies in Western contexts often featured triangular or dispersed spatial layouts. This discrepancy highlights the symbolic inadaptability of existing art therapy frameworks across cultural contexts. Moreover, the majority of current research focuses on short-term effects, with limited longitudinal data to support claims of sustained therapeutic impact.

To address these limitations, this study introduces an innovative framework that integrates embodied cognition theory with culturally rooted visual metaphors. Specifically, traditional Chinese ink painting techniques were incorporated into the intervention. Rendering (渲染, *xuan ran*) involves layering diluted ink in gradual washes to create depth, softness, and tonal transitions that convey atmosphere and emotional nuance. Blank leaving (留白, *liu bai*), by deliberately preserving unpainted space, emphasizes contrast, balance, and openness, enabling viewers to project meaning into the composition. Students were further encouraged to integrate traditional cultural symbols (e.g., the lotus flower representing purity) into their artwork. To enrich this process, an adjustable light-and-shadow device was designed to simulate the ink-painting contrast between emptiness and fullness, thereby creating an immersive environment inspired by the Eastern aesthetic principle of harmony between humans and nature (天人合一, *tian ren he yi*). These culturally grounded strategies fostered deeper emotional engagement and resonance in social interaction. In parallel, culturally sensitive evaluation tools—such as a “symbolic pattern integrity score”—were developed to interpret artworks within a cross-cultural therapeutic framework. Collectively, these methods establish a scientifically rigorous and culturally adaptive model for autism intervention, integrating embodied cognition with localized symbolic systems.

### 2.5 Research design

This study adopted a mixed method design (Lee, 2019), integrating embodied activities and cultural symbols based on the EC-DT model, combined with randomized controlled trials (RCT) and interpretive phenomenological analysis (IPA), and conducted 2 case studies and 60 group experiments, respectively. 60 autistic students (6–19 years old) were randomly divided into an intervention group (EC-DT model, *n* = 30) and a control group (conventional training, *n* = 30).

### 2.6 Participants

Participants were recruited from a special education institution using a convenience sampling method. Inclusion criteria were as follows: (1) a confirmed diagnosis of autism spectrum disorder (ASD) according to the DSM-5 criteria; (2) a score of ≥30 on the Childhood Autism Rating Scale (CARS), indicating moderate to severe symptom severity; (3) artwork evaluated independently by two certified art therapists using the Self-Portrait Drawing Assessment (SPD) and the Diagnostic Drawing Series (DDS), both of which exhibited typical ASD-related drawing characteristics (e.g., prioritization of detail, avoidance of eye contact); and (4) a Wechsler Intelligence Scale for Children – Fourth Edition (WISC-IV) score between 50 and 85, indicating mild to moderate intellectual functioning. Informed consent was obtained from the participants’ legal guardians. Exclusion criteria included: (1) the presence of severe physical illnesses; and (2) having received other structured therapeutic interventions within the preceding 3 months.

### 2.7 Assessment tools

This study employed a multidimensional assessment system comprising both quantitative and qualitative evaluations. Quantitative assessments included the Goal Attainment Scaling (GAS); Positive and Negative Syndrome Scale (PANSS), which measured the severity of core autism-related symptoms; the Tennessee Self-Concept Scale (TSCS), which evaluated participants’ self-perception across multiple domains; and the General Quality of Life Inventory-74 (GQOL-74), which assessed physical, psychological, and social well-being. In addition, two standardized art-based tools, the Self-Portrait Drawing (SPD) and the Diagnostic Drawing Series (DDS), were used to analyze visual features associated with autistic traits. Qualitative data obtained through semi-structured interviews and drawing analysis, provides insight into the participants’ emotional expression and social behavior. Details of the quantitative instruments and implementation procedures are summarized in Table 1.

**Table 1:** Assessment tools.

Tool name	Assessment dimension	Reference source	Implementation specification
SPD	Scores from three dimensions (0-5 points): line smoothness, detail integrity, and color use.	Martin (2008), Papangelo et al. (2020)	Double-blind independent scoring, high-definition scanning archive
DDS	Scoring from 0-10 points based on emotional projection patterns (such as trees symbolizing security) and composition integrity.	Cohen et al. (1994)	Timed completion, expert blind evaluation
PANSS	Positive and negative symptoms, general psychopathology	Kay et al. (1987)	Double-blind rating, Likert 5-point method
GAS	Social adaptive functioning and symptom severity	Endicott et al. (1976)	Standardized behavioral observation records
GQOL-74	Physiological, psychological, social and environmental functions	Aalbers et al. (2020), Jia et al. (2023)	To be filled in by the examiner, anonymous
TSCS	10 factors including physical self, moral self, etc.	Crites (1965)	Structured interview, multiple points scoring

The evaluation process was structured as follows:

1. Baseline Assessment (Weeks 1–2): Participants completed pre-intervention evaluations using the PANSS, Goal Attainment Scaling (GAS), and other standardized scales. Baseline drawings were collected using the Self-Portrait Drawing Assessment (SPD) and the Diagnostic Drawing Series (DDS).
2. Intervention Phase (Weeks 3–11): The intervention group received painting-based therapy grounded in the Embodied Cognition Drawing Therapy (EC-DT) model, administered twice per week for 90–120 min per session. The control group continued with routine rehabilitation training without exposure to art-based intervention.
3. Post-Intervention and Follow-Up (Week 12 and 3-Month Follow-Up): Participants completed post-tests of the same scales. Additional measures included a parental satisfaction survey and comparative analysis of drawings to evaluate emotional and symbolic changes.

### 2.8 Experimental process

**Figure 1:**
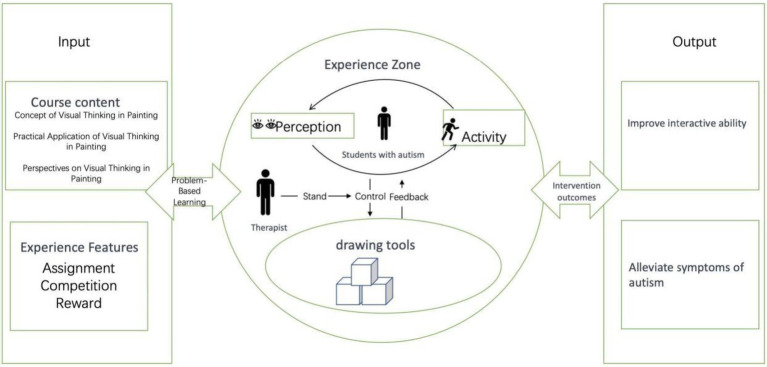
EC-DT teaching model based on embodied cognition.

This study adopted a randomized controlled trial design in which participants were randomly assigned to either the intervention group or the control group. The intervention was grounded in the Embodied Cognition Drawing Therapy (EC-DT) model, a novel approach that integrates body-based engagement with culturally embedded artistic expression (Figure 1). Rather than relying on isolated cognitive tasks, the EC-DT model emphasizes full-body participation through tactile painting and body imitation, while also incorporating traditional Chinese patterns that carry deep symbolic meaning. Throughout the sessions, participants were guided to engage in multisensory experiences—combining painting, music, and movement—which not only enriched their emotional expression but also fostered synchronized group interaction and peer connection. The therapeutic process unfolded in three structured stages, each designed to reflect both the developmental needs of the participants and the therapeutic objectives of the intervention.

The implementation began with a pre-test phase during weeks 1 to 2, in which a multidimensional baseline assessment was conducted to establish a comprehensive understanding of each participant’s clinical and functional status. We used standardized PANSS diagnostic interviews, while drawing ability was evaluated through SPD self-portraits and DDS drawing tasks conducted in a controlled environment. Functional levels were observed and rated by therapists using the Goal Attainment Scaling (GAS) method, and quality of life was measured via the GQOL-74 questionnaire, which participants completed with the support of trained experimenters. In addition, structured interviews based on the Tennessee Self-Concept Scale (TSCS) were conducted to assess self-perception. Each participant’s painting characteristics—such as color preferences, compositional tendencies, and motor patterns—were documented in individualized files, and all initial artworks were high-resolution scanned (300 dpi) for digital archiving. Parents were also asked to complete the Child Behavior Checklist (CBCL), contributing additional data on behavioral tendencies from the home environment.

The intervention phase spanned weeks 3 to 11 and followed a progressive structure divided into three stages, each with clear objectives and task sequences (as detailed in Table 2). In the first stage (weeks 3–5), the focus was on basic skill building, using tasks like graffiti exploration and simple shape imitation to help participants gain familiarity with materials and build a therapeutic alliance. The second stage (weeks 6–8) introduced emotional expression training, engaging students in drawing emotional faces and life scenes to foster emotion recognition and expression through visual and tactile modalities. Finally, in the third stage (weeks 9–11), social interaction application tasks such as collaborative painting and themed group creations were introduced to promote communication, cooperation, and symbolic interaction among peers. Each intervention session lasted 90 to 120 min and followed a consistent structure: warm-up activities, thematic painting, and group discussion. Sessions were conducted twice weekly using standardized tools and painting materials to ensure consistency across participants. Full dual-camera video recordings were made of every session, and therapists filled out detailed Process Record Forms documenting behavioral responses, participation levels, and any problem behaviors observed. Weekly supervision meetings were held to review these records and ensure strict adherence to intervention protocols and fidelity.

**Table 2:** Group painting art intervention painting tasks chart.

Theme	I	II	III	IV	V	VI
Graffiti	Build interest in painting and reduce unfamiliarity with the tools	Basic point training; improve fine motor control of hands	Basic line training; enhance movement coordination and emotional catharsis	Graphics basis; develop geometric figure recognition ability	Color basis; master the relationship between color and emotion	Color basis; enhanced multi-color collaborative application
Portrait	Any portrait; observing initial self-representation abilities	Same sex; promoting gender identity and social referencing	Opposite sex; expand social cognition	Other people; enhance empathy and observation	Self; reconstructing the self-concept	Future self; stimulate a sense of hope and planning skills
Scene	Any scene; assessing spatial perception and imagination	Studio; judgment of safety in the treatment environment	Home; emotional projection of family relationships	Play or park; social situation understanding	Living space; daily life adaptability	Ideal space; inner needs and desires
Feelings	Any mood; natural expression of emotions	Fear or worry, venting negative emotions	Peace and comfort; emotional regulation expression	Satisfaction or joy; positive emotional experience	Happiness and love; build emotional connection	Realistic feelings; cognition and emotional expression
Co-painting	Any painting; initial interaction behavior	Assignment of tasks; division of labor and rule compliance	Integration of content; teamwork and communication	Integration of color; conflicting color choices	Group work; social reinforcement of shared goals	Rehabilitation life; real social scene expression

Meanwhile, the control group participated in conventional Applied Behavior Analysis (ABA) therapy, which was delivered five times per week in 45-min sessions by special education professionals. Artistic activities were deliberately excluded to maintain the specificity of the experimental intervention. Both groups operated within the same school environment to ensure ecological validity, and all intervention-related personnel were trained under unified standards to minimize procedural variance.

Following the intervention, a post-test phase was conducted during weeks 12 to 13, repeating all baseline assessments using identical tools and procedures. To ensure objectivity, all evaluations were performed by professionals blinded to group assignments. Beyond the repeated clinical and behavioral scales, a comparative artwork analysis was conducted. Three certified art therapists, also blind to participant groupings, used the Painting Development Assessment Scale to quantitatively score the participants’ drawings in terms of emotional expressiveness, symbolic complexity, and developmental markers. Supplementary evaluations included a parent satisfaction survey using a 5-point Likert scale, qualitative therapist reports, and structured interviews with both parents and teachers to assess perceived changes in participants’ emotional and social functioning.

A follow-up evaluation was conducted 3 months later. Data integrity and research quality were safeguarded through weekly fidelity supervision, consistent documentation using the REDCap system, and strict adherence to ethical standards under the guidance of the CONSORT statement. All assessors underwent inter-rater reliability training, achieving a Kappa coefficient above 0.75, thereby ensuring scoring consistency across time and raters. The EC-DT Course Manual, which outlines every intervention procedure in detail, served as a standardized guide for implementation, and a contingency withdrawal mechanism was established to protect participant safety. Taken together, this rigorously controlled, and richly contextualized implementation process not only ensured the scientific robustness of the study but also highlighted the practical viability and cultural adaptability of the EC-DT model in autism intervention.

### 2.9 Intervention program

**Figure 2:**
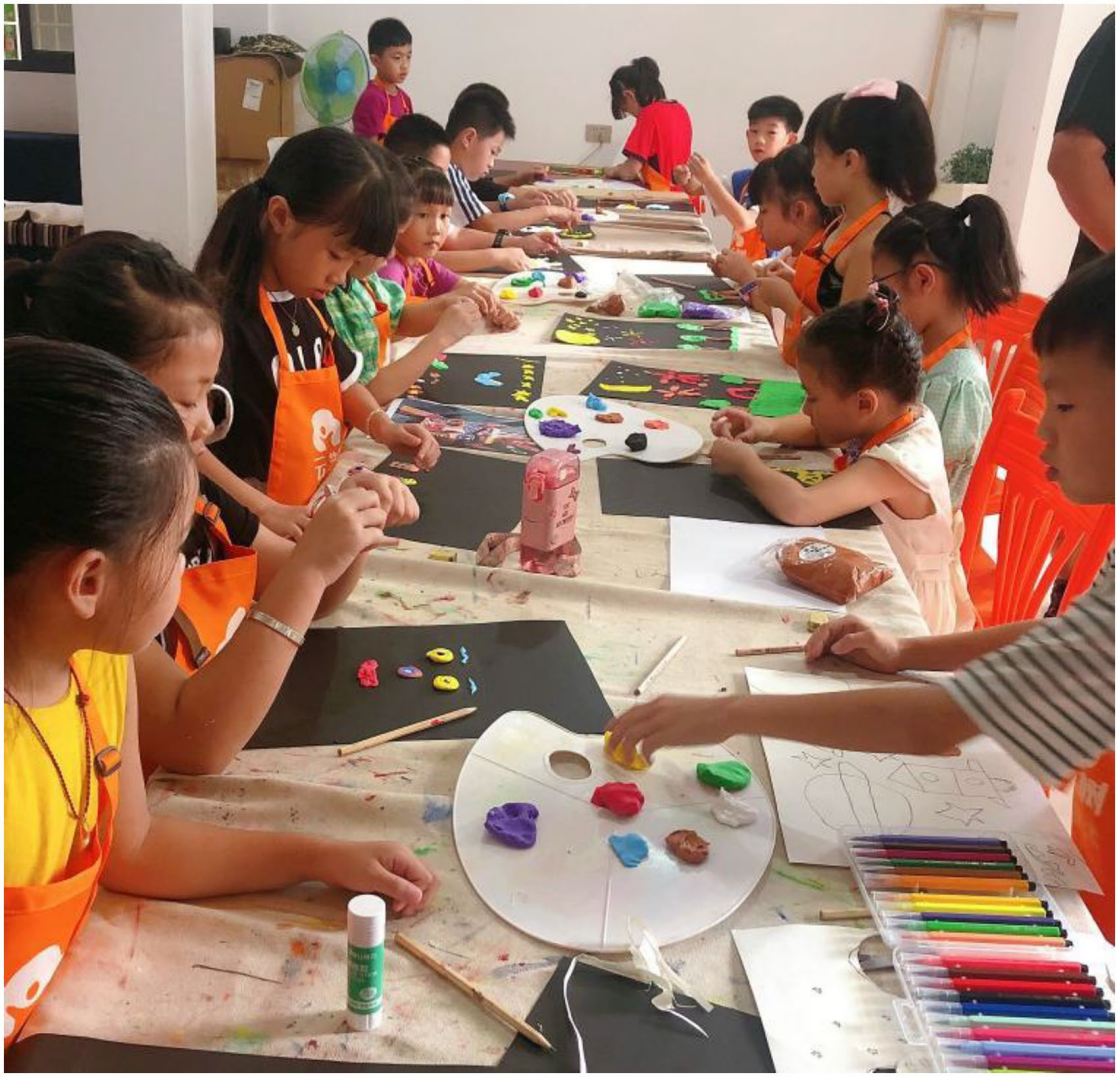
Students’ collective painting experience.

To enhance social engagement and mutual understanding between autistic students and their typically developing peers, this study implemented a structured group painting program grounded in embodied cognition theory and emotional expression through art. A total of 60 participants—30 autistic students and 30 typically developing peers—were age-matched and divided into small groups (Figure 2). Under the guidance of a trained art therapist, each group participated in a three-step standardized painting process designed to break social barriers, facilitate emotional expression, and promote cooperative interaction.

The session began with an ice-breaking activity in which each participant was invited to introduce themselves using a single word that best represented their current mood or personality. This stage, limited to 10 min, provided a gentle and accessible entry point into social interaction, especially for students with initial social anxiety. With the encouragement and modeling of peers and therapists, even those exhibiting reluctance or phobic tendencies gradually participated, laying a foundational level of comfort and openness necessary for deeper engagement later in the session.

**Figure 3:**
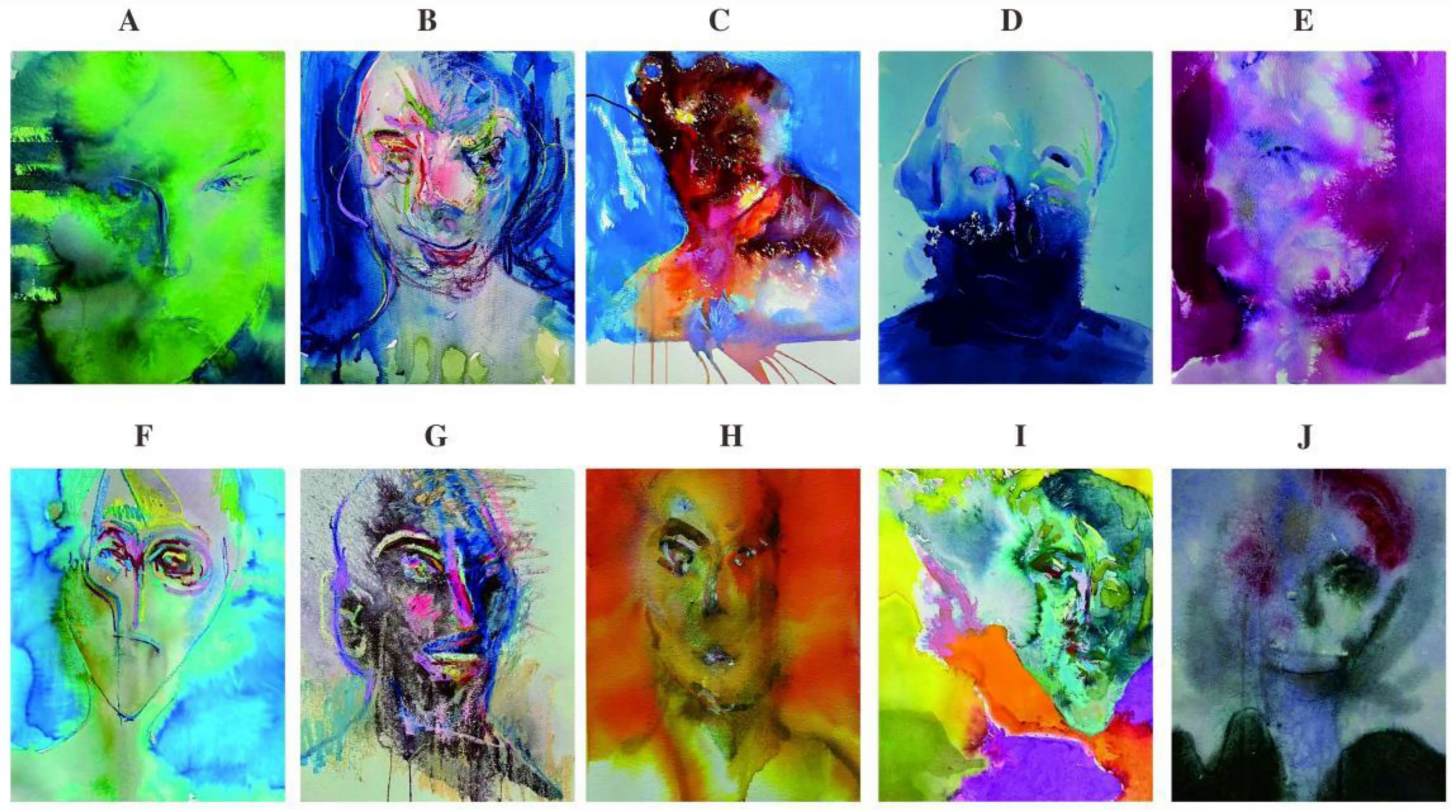
Representative Chinese subjects’ specimen portraits. Subject **A**’s portrait looks at the painting, does not speak, and does not communicate. Subject **B**’s portrait avoids the eyes, and the portrait features are unclear. Subject **C** repeatedly wipes the painting and tears up the finished product. Subject **D** repeatedly scribbled, and the specimen portrait is blue. Subject **E** is repeatedly altered, and the portrait features are unclear. Subject **F**’s specimen portrait has stereotyped behavior and exaggerated eyes. Subject **G** vents his negative emotions through graffiti. Subject **H**’s portrait outline is repeatedly modified, avoiding the eyes, smearing color blocks, and the facial features are unclear. Subject **I**’s portrait became smudged and blurred, as he repeatedly smeared and rubbed the edges with his fingers before the paint had dried. Subject **J**’s specimen portrait is repeatedly altered, and the facial features of the portrait are incomplete.

Following this, the portrait painting task was introduced. Students were provided with a consistent set of painting materials and given 20 min to create an imaginative self-portrait. Rather than focusing on realism or technique, the task emphasized creativity and individual expression. To further stimulate embodied interaction with the medium, the therapist introduced dynamic techniques—such as rotating the paper, altering brush grips, or using non-dominant hands—to encourage playful experimentation and sensory engagement. This phase of the session not only revealed participants’ artistic tendencies but also helped bypass verbal limitations, especially for those with language delays or social inhibition (Koppitz, 1966). The resulting artworks often carried symbolic representations of identity, mood, and perception, serving as visual narratives for each participant’s internal state (Figure 3).

The third phase focused on emotional expression and group integration. Autistic students frequently displayed tension or avoidance behaviors in their drawings—for instance, repetitive motifs like circles, or a reluctance to depict eyes or faces, reflecting their social anxiety and need for emotional safety. The therapist actively modeled the use of expressive lines, abstract forms, and emotionally resonant colors, guiding students to translate their internal feelings onto the page. Over the course of the 90-min session, the group gradually entered a more cohesive and emotionally responsive state. Through demonstration, shared commentary, and supportive dialogue, even initially withdrawn participants began to interact with others’ artworks and engage in reciprocal expression. The session concluded with verbal affirmations from the therapist, reinforcing group cohesion and encouraging continued participation in future activities.

A noteworthy finding of the study was the significant correlation between the color preferences of autistic participants and their behavioral profiles. As illustrated in Figure 3, students who predominantly used blue or green often presented as emotionally withdrawn, solitary, and self-focused traits more commonly observed in male participants. In contrast, those who gravitated toward red or orange exhibited higher emotional arousal, impulsivity, and expressive energy, with a notable prevalence among female students. Yellow, frequently associated with dependency and milder symptoms, was most often selected by participants who demonstrated relatively stable interpersonal responses and adaptive behavior, also more frequently female. These patterns resonate with the observations of Lev-Wiesel and Shvero (2003), supporting the argument that color serves as a vital, non-verbal medium through which autistic individuals externalize emotional states and internal conflicts.

#### 2.9.1 Case analysis: painting therapy process of subject A

Subject A (No. 29) is an 8-year-old male student, born in September 2017 and currently enrolled in the first grade. His mother is a full-time homemaker, and his father is an engineer whose frequent work-related travel results in limited daily involvement in family life. Subject A was clinically diagnosed with moderate autism spectrum disorder (ASD) at the age of 7 and exhibits characteristic challenges in social interaction, verbal communication, and interest development. The following is a staged analysis of his engagement in the EC-DT model painting therapy, revealing changes in his behavioral patterns, cognitive representations, and emotional responses through drawing.

##### 2.9.1.1 Stage 1: establishing attachment and environmental adaptation

**Figure 4:**
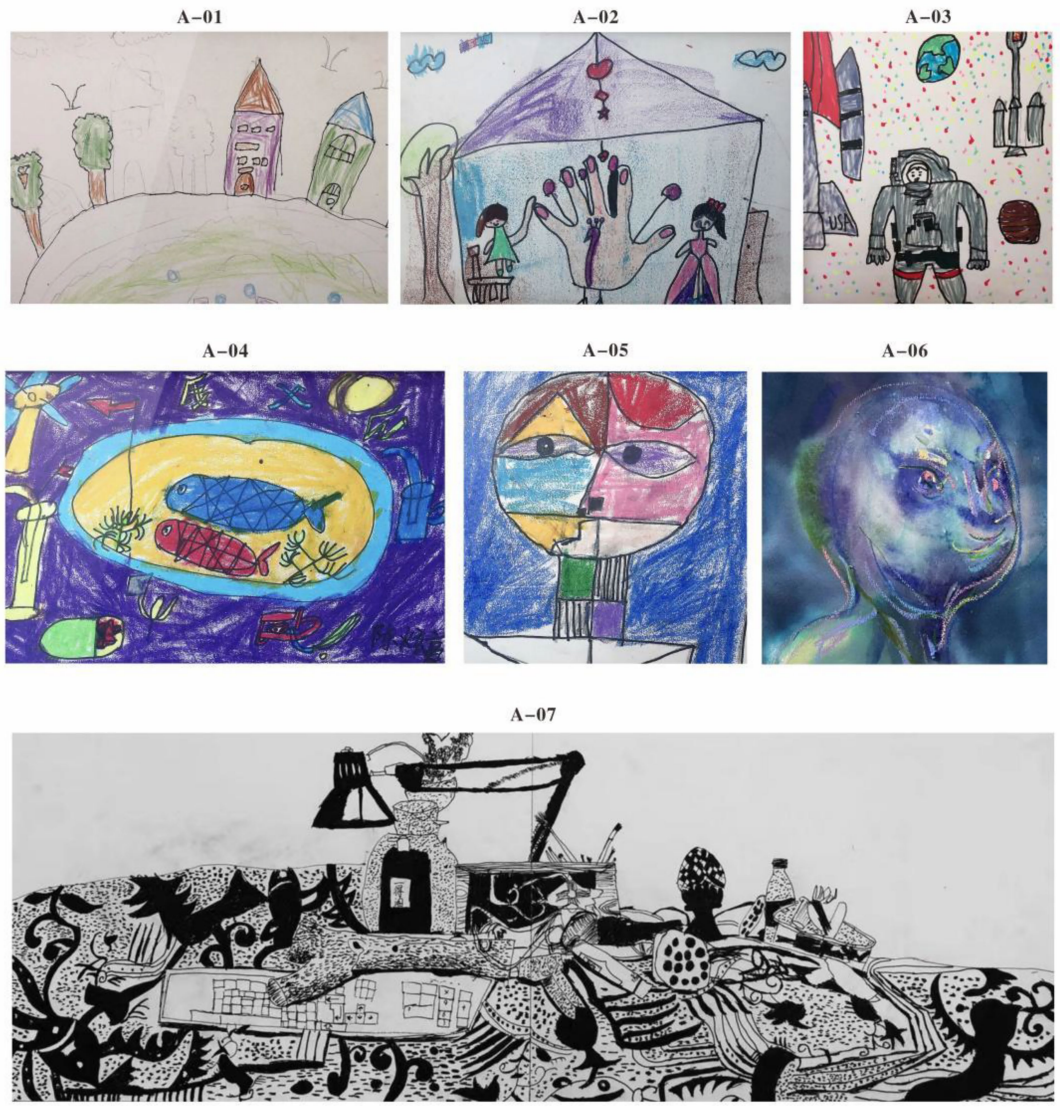
The implementation process of the painting art intervention of subject A. **(A-01)** Basic shape drawing method, **(A-02)** graffiti drawing method and expression, **(A-03)** free expression of geometric patterns I, **(A-04)** subject A’s free expression of geometric patterns II, **(A-05)** color graffiti self-portrait, **(A-06)** color card self-portrait, **(A-07)** concrete description of real scenes.

In the initial sessions, the therapeutic goal was to create a secure and predictable environment in which Subject A could begin to engage. This stage involved simple line-drawing games and geometric figure tasks, where circles and triangles were introduced as metaphorical representations of houses and people. Subject A demonstrated a cautious but observable adaptation to the treatment setting. While he maintained an absence of eye contact—a common trait in children with ASD—he did not display avoidance or resistance behaviors (Figures 4A-01,A-02). His silent but cooperative participation suggested an emerging sense of psychological safety, which is crucial for deeper emotional work in later phases.

##### 2.9.1.2 Stage 2: self-exploration through free drawing

As the sessions progressed, Subject A was encouraged to engage in free drawing, leading to spontaneous creations such as robots and marine life. These imaginative themes reflect both a cognitive preference for mechanical systems and a fascination with nature’s patterns—two recurring motifs among children on the spectrum. However, an interesting observation emerged: his drawings frequently exhibited open or fragmented contours, lacking closure in forms that typically define object boundaries (Figures 4A-03,A-04). This aligns with findings from Baron-Cohen et al. (1985), which describe a characteristic cognitive style in autism involving difficulties with gestalt processing and a tendency to focus on local details over global coherence.

##### 2.9.1.3 Stage 3: social representation and reinforced engagement

With increasing familiarity and targeted guidance, Subject A began to demonstrate improved representational capacity. Using embodied strategies such as mirror observation and illustrated card prompts, he gradually succeeded in drawing facial features—a task previously avoided or rendered symbolically (Figures 4A-05,A-06). This shift reflects progress in both motor planning and self-recognition, suggesting that embodied action supported his capacity to internalize and reproduce human features. The emergence of facial elements in his drawings not only indicates enhanced perceptual integration but also marks a meaningful step toward the development of social cognition.

##### 2.9.1.4 Stage 4: embodied cognition and episodic memory integration

The final stage of therapy focused on the reconstruction of episodic memory through embodied drawing. When prompted to represent real-life scenes, such as family meals or school activities, Subject A displayed significant difficulty in spatial differentiation. Rather than articulating distinct environments or spatial relationships, he tended to centralize objects and reduce them to basic geometric forms, such as circles and squares, while investing considerable effort in surface-level details (Figure 4A-07). This phenomenon reflects a key feature of the Weak Central Coherence (WCC) theory, wherein individuals with ASD prioritize fragmentary details over holistic structure. Subject A’s drawing patterns suggest an episodic memory system shaped by sensorimotor salience rather than contextual sequencing, highlighting the potential of embodied artistic interventions to reorganize fragmented perceptual experiences gently.

Through the progressive stages of this intervention, Subject A moved from cautious engagement to meaningful self-expression and social representation. His visual language evolved from abstract and disconnected forms to increasingly coherent depictions of self and others. The therapy not only revealed the cognitive architecture underlying his drawing behaviors but also served as a non-verbal bridge for emotional expression and social connection, core goals of the EC-DT model.

#### 2.9.2 Case analysis: painting therapy process of subject B

Subject B (No. 17) is an 18-year-old female high school senior, born in June 2007. Her parents work as ordinary company employees, and she was diagnosed with mild autism spectrum disorder (ASD) at the relatively late age of 15. B exhibits typical traits associated with high-functioning ASD, including pronounced social withdrawal, preference for solitude, lack of facial expressiveness, and poor attentional control. Her condition worsened during the COVID-19 pandemic due to prolonged social isolation, triggering a significant increase in anxiety and emotional instability. This case study documents how a five-stage painting therapy intervention facilitated improvements in her emotional regulation, self-perception, and interpersonal awareness.

##### 2.9.2.1 Stage 1: emotional catharsis through line expression

**Figure 5:**
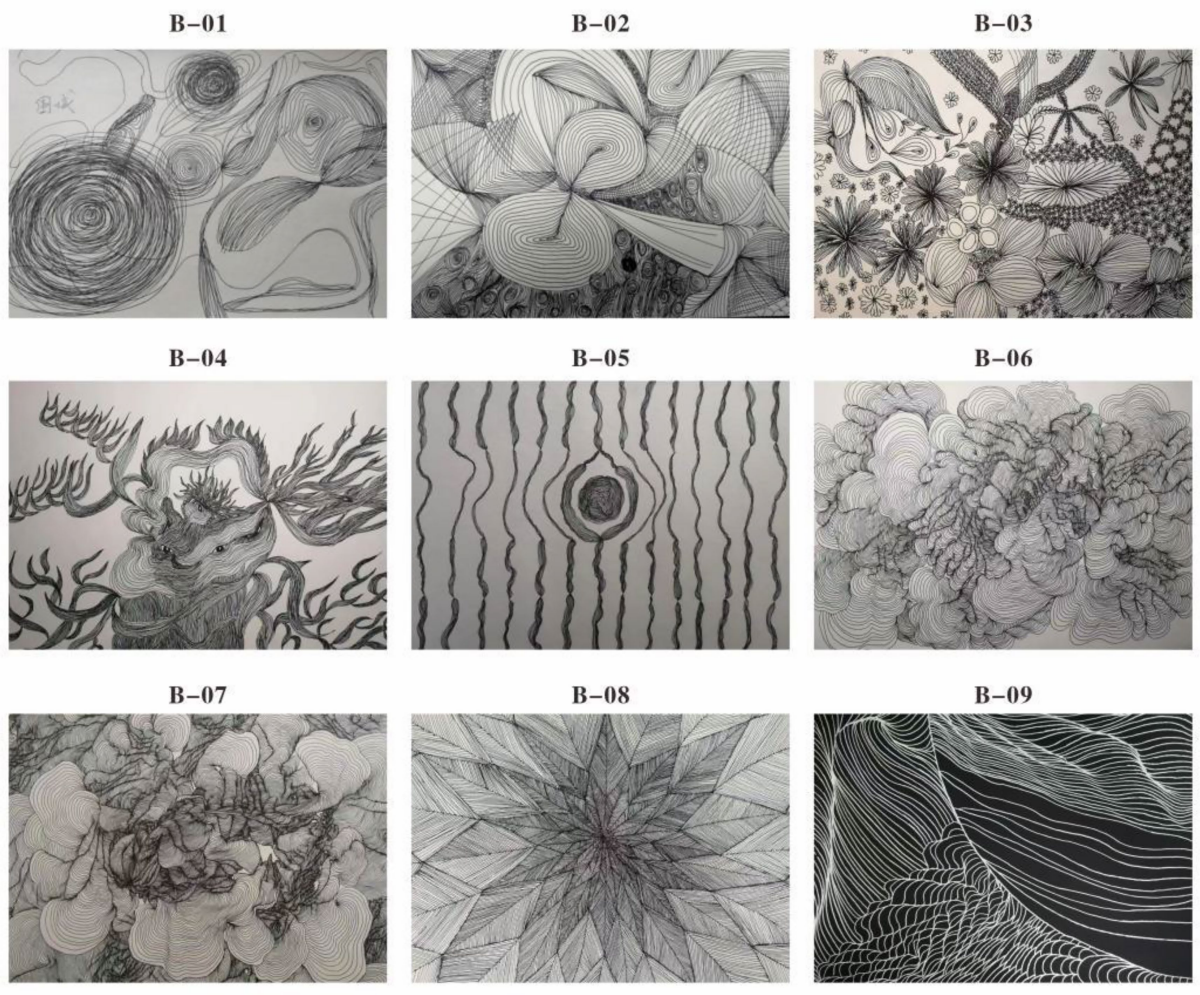
Cognitive painting art therapy I of subject B. **(B-01)** Chaotic line expression I of subject B **(B-02)** chaotic line expression II **(B-03)** results of imitating line drawing training **(B-04)** finding regular lines representing happiness **(B-05)** emotional expression **(B-06)** Expression of emotional stability line **(B-07)** expression with non-intersecting wavy lines **(B-08)** regular linear representation **(B-09)** curvilinear representation.

In the initial phase, Subject B’s drawings were dominated by dense, disordered, and overlapping lines, with one work explicitly titled “Siege,” suggesting a sense of psychological entrapment and defensive self-isolation (Figures 5B-01,B-02). This aligns with Lev-Wiesel and Shvero (2003) view that chaotic line work often symbolizes internal emotional turmoil and barriers in social communication. The therapist guided B through freeform line exercises, allowing her to discharge internal anxiety through unstructured yet intentional movement. As therapy progressed, she began to experiment with more open and rhythmic line arrangements, symbolically reflecting a shift in emotional tone. In a key turning point, B depicted “sunlight” using curved, bright lines (Figure 5B-03), marking a perceptible reduction in anxiety and a willingness to re-engage with the external world (Koppitz, 1966).

##### 2.9.2.2 Stage 2: line imitation and structured self-exploration

In this stage, Subject B transitioned from chaotic expression to imitative practice. She was encouraged to replicate regular line patterns, beginning with straight lines and gradually incorporating arcs and waves (Figure 5B-04). Her early attempts were marked by uneven spacing and abrupt direction changes, indicating internal tension and difficulty with sensorimotor planning. However, over repeated sessions, B gained increasing control over her line execution. The emergence of orderly, continuous lines mirrored improvements in emotional regulation and cognitive coherence. This stage supports Baron-Cohen et al. (1985) assertion that rhythmic visual-motor tasks can foster affective stability and reinforce internal sense of order in adolescents with neurodevelopmental challenges.

##### 2.9.2.3 Stage 3: embodied perception and multimodal integration

During the third phase, Subject B began integrating multiple sensory modalities. For the first time, she mixed multiple colors with a deliberate aesthetic intention (Figure 5B-05). The therapist introduced tactile painting tools—such as textured rollers and finger-painting pads—to deepen her engagement with bodily sensation and promote embodied awareness. The fluidity and composition of her lines improved, and her drawings began to exhibit greater visual balance. Nevertheless, B consistently avoided depicting complex objects or spatially differentiated scenes, suggesting residual deficits in spatial cognition and gestalt integration—cognitive traits consistent with the Weak Central Coherence theory (Baron-Cohen et al., 1985).

##### 2.9.2.4 Stage 4: physiological reactions and symbolic displacement

In a period of intensive intervention, B practiced repetitive line patterns—particularly straight lines and ripples—as a form of meditative engagement (Figures 5B-06–B-09). This repetitive visual-motor task effectively reduced her daily emotional outbursts (from five episodes to two), indicating improved self-regulation. However, during this phase, she reported transient physiological discomfort, including dizziness and nausea. Concurrently, her artwork began to incorporate ambiguous symbolic imagery resembling visual hallucinations—an effect possibly linked to sensory overload or synesthetic misinterpretation (Hammer, 1968). Despite these reactions, her parents noted that B’s behavioral predictability improved, and her adaptability in routine social settings (e.g., classroom, family events) showed noticeable enhancement.

##### 2.9.2.5 Stage 5: symbolic expression and cultural reconnection

**Figure 6:**
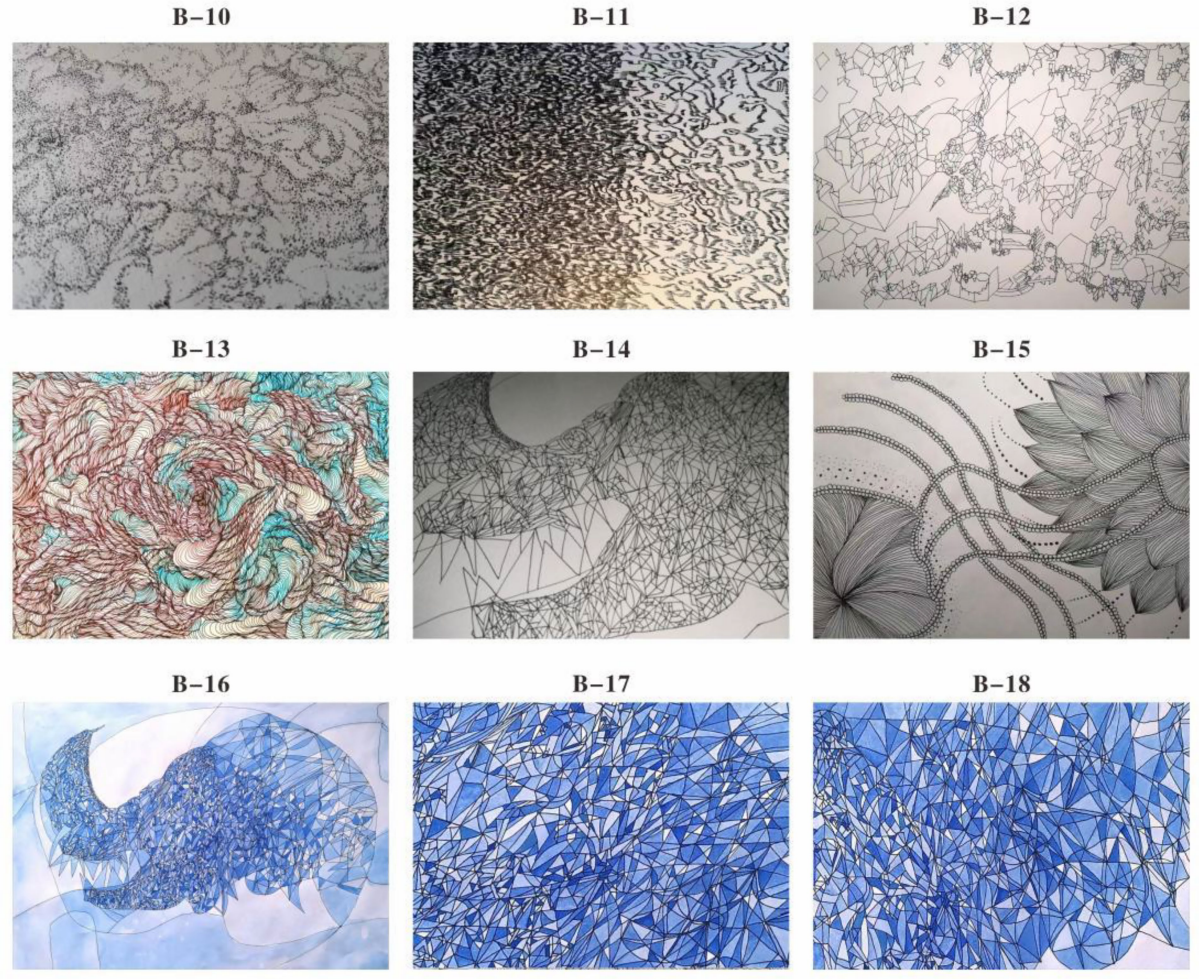
Cognitive drawing art therapy II for subject B. **(B-10)** Dashed linear representation of subject B **(B-11)** dense linear representation **(B-12)** closed linear representation **(B-13)** Linear representation using color perception **(B-14)** close-up graphic representation **(B-15)** linear representation in emotional stability **(B-16)** color representation of closed shapes I **(B-17)** color representation of closed shapes II **(B-18)** color training of closed shapes.

**Figure 7:**
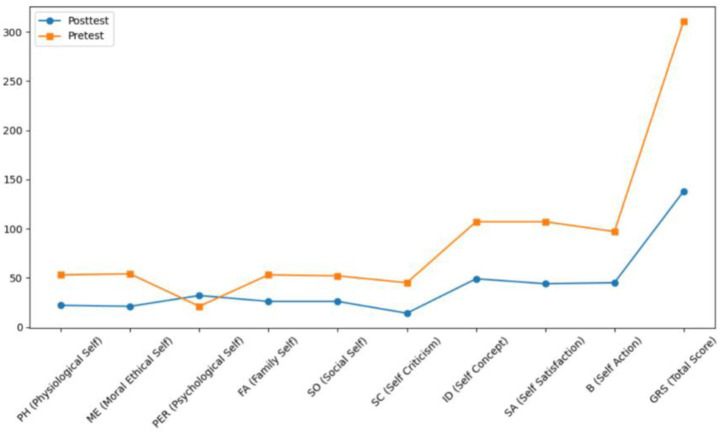
Self-concept assessment results of subject A before and after intervention.

**Figure 8:**
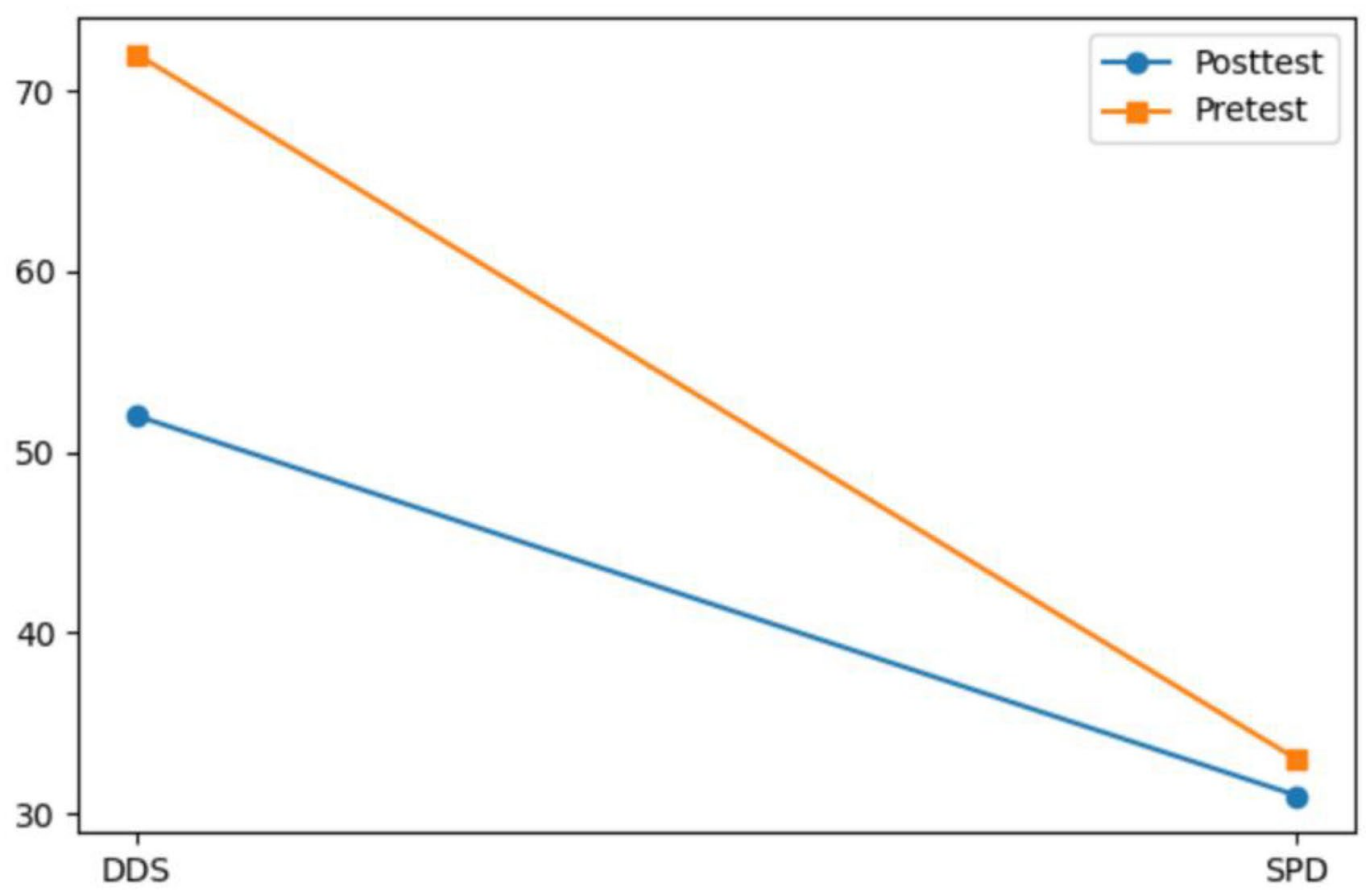
Drawing evaluation results of subject A before and after intervention.

**Figure 9:**
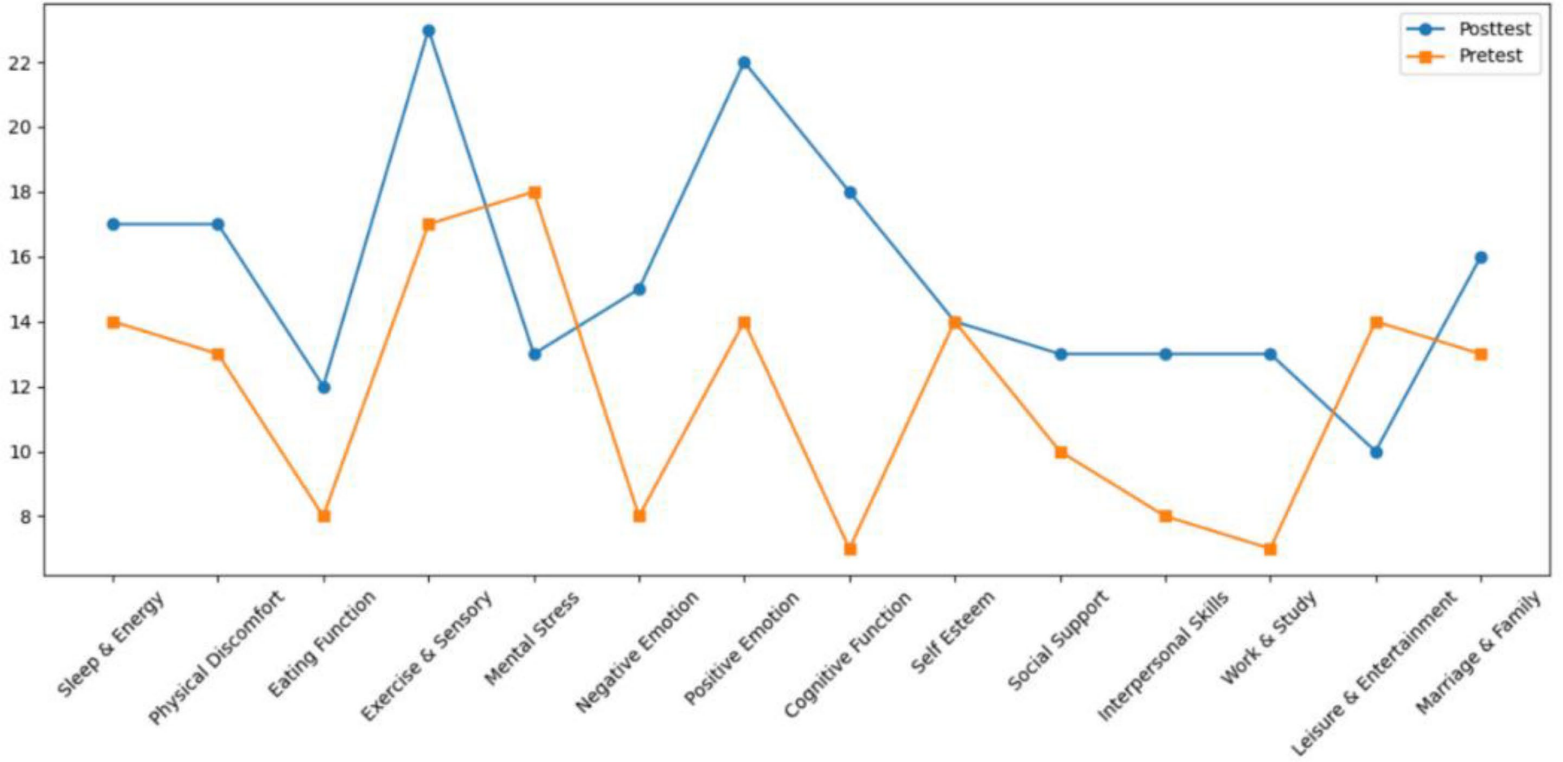
Quality of life of subject A before and after intervention.

**Figure 10:**
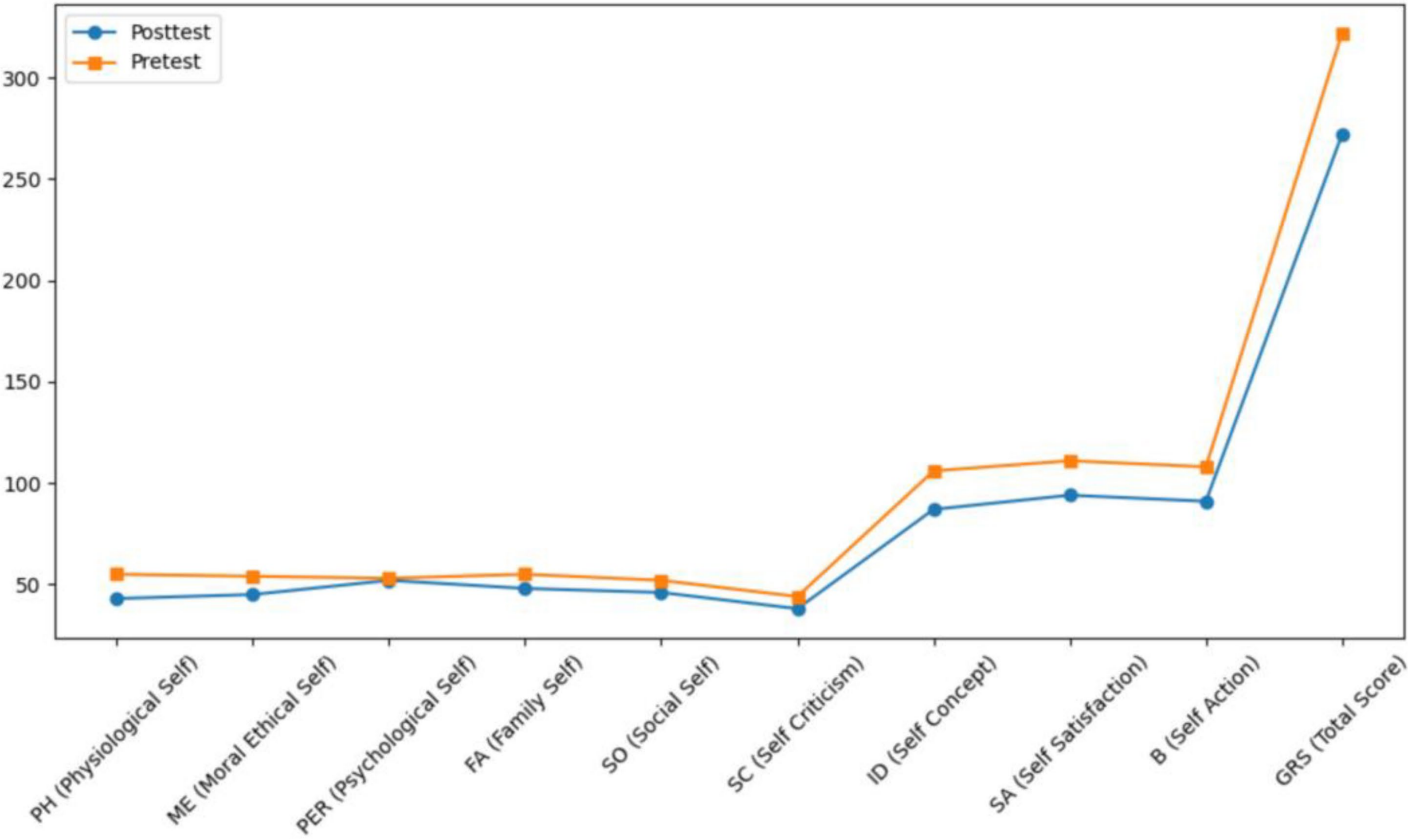
Self-concept estimation results of subject B before and after intervention.

**Figure 11:**
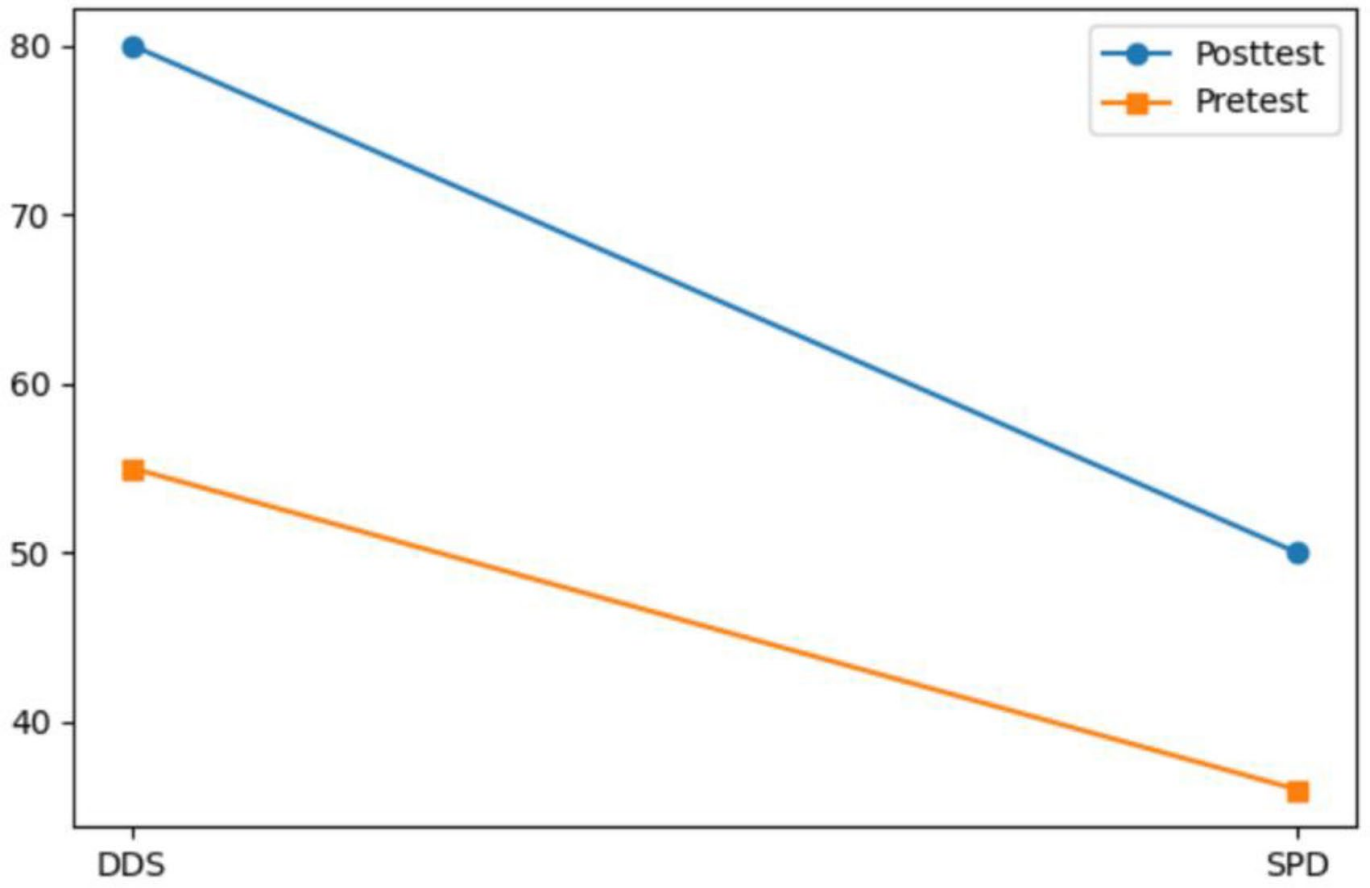
Results of subject B’s painting evaluation before and after the intervention.

**Figure 12:**
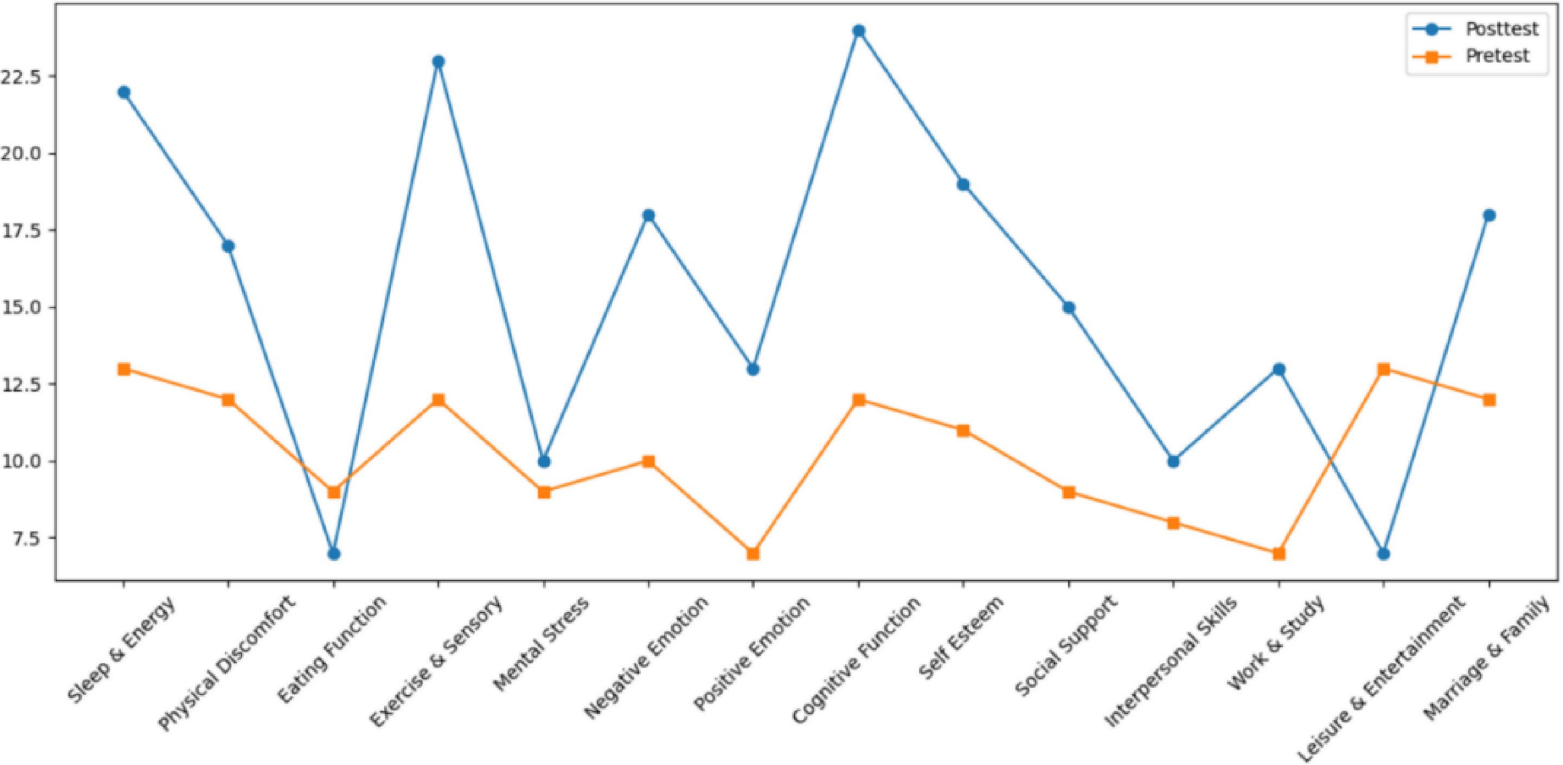
Quality of life of subject B before and after intervention.

In the final stage, Subject B’s focus shifted toward symbolic and metaphorical representation of self and society. She began exploring identity and relational positioning using closed shapes (e.g., nested circles, angular borders) and deliberate color infill techniques (Figures 6B-13–B-18). One particularly significant image was a “sawtooth wave” drawn in bold, dark hues—an abstract representation of emotional fluctuation and tension release (Figure 6B-18). She also incorporated traditional East Asian motifs, such as auspicious clouds and repetitive wave patterns, to express belonging and harmony. These visual symbols echoed the metaphor of “和” (harmony) in Eastern aesthetics, as discussed by Cui and Wang (2022). After completing this phase, B’s self-critical tendencies visibly diminished, and she was more willing to participate in group discussions, suggesting a strengthened sense of self-acceptance and emotional resilience.

Both Subject A and Subject B completed a nine-week group painting intervention, with comprehensive assessments conducted before and after the program, including psychological evaluation, artistic performance analysis, self-concept measurement, and quality of life assessment. Subject A demonstrated significant reductions in anxiety, depression, and paranoid symptoms, with a lower total score on psychological assessments and improved behavioral functioning as indicated by a higher GAS score. His painting assessment score decreased, suggesting a shift from chaotic to more organized visual expression, while notable improvements were observed in emotional well-being, cognitive functioning, and social adaptability. Subject B showed a marked decline in self-critical tendencies, along with enhanced self-concept and self-satisfaction. Her painting assessment score also decreased, reflecting improved emotional regulation and expressive clarity. Furthermore, she exhibited significant gains across multiple quality of life domains, including sleep quality, energy levels, physical comfort, eating behavior, emotional stability, cognitive functioning, social support, interpersonal communication, academic engagement, and leisure participation. These outcomes collectively support the efficacy of painting-based interventions grounded in embodied cognition theory in promoting emotional, cognitive, social, and physical well-being in individuals with autism (see Figures 7–12).

## 3 Data processing and experimental results

To ensure comparability of the evaluation of the intervention effect, this study conducted a baseline assessment of mental symptoms and overall functional level in the intervention group and the control group before the intervention. The assessment tools include GAS and PANSS, covering multiple dimensions such as emotions, behaviors, thinking, and social functions. The GAS scale is used to assess the overall performance of individuals in psychological, social, and occupational functions. The higher the score, the better the function. The PANSS scale is further refined into multiple sub-dimensions such as positive symptoms (such as excitement, impulsive behavior), negative symptoms (such as withdrawal, emotional indifference), thinking disorders, non-reaction syndrome, depressive symptoms, and active symptom groups.

As shown in Tables 3, 4, before the intervention began, there were no significant differences between the intervention and control groups in terms of overall functioning and psychiatric symptoms, ensuring the comparability of both cohorts. Following the 9-week drawing-based intervention, the intervention group demonstrated a substantial improvement in their overall GAS score—from a baseline average of 59.80 (SD = 2.52) to 90.80 (SD = 2.27). In contrast, although the control group also showed progress (from 55.90 to 88.70), the magnitude of improvement was less pronounced. This suggests that beyond the natural developmental course or general supportive training, the EC-DT intervention had an additional and targeted benefit in enhancing psychosocial functioning. On the PANSS scale, marked improvements were also observed in the intervention group across several symptom domains. Negative symptoms, including emotional flatness and limited verbal communication, decreased from an average of 11.10 (SD = 1.37) to 5.57 (SD = 0.86), while cognitive disorganization symptoms dropped from 5.80 (SD = 1.08) to 2.02 (SD = 1.12), reflecting notable gains in emotion regulation, expressive fluency, and internal thought structure. In the PANSS scale, the intervention group showed varying degrees of symptom relief in multiple sub-dimensions after the intervention. For example, in the negative symptom score, the intervention group’s score decreased from 11.10 (SD = 1.37) to 5.57 (SD = 0.86), and in terms of thought disorder symptoms, it decreased from 5.80 (SD = 1.08) to 2.02 (SD = 1.12), indicating that there was a significant improvement in emotional indifference, language delay, and cognitive processing after the intervention.

**Table 3:** Results of the pre-psychiatric evaluation of the intervention and control groups.

Gross rating	Groups	Mean	Min	Max	Std	Skewness	Kurtosis
GAS score	Intervention group	59.80	55.00	63.00	2.52	0.01	0.06
	Control group	55.90	53.00	59.00	1.81	0.23	0.01
Positive and negative symptom scale							
Positive scale score	Intervention group	15.80	13.00	19.00	1.72	0.22	−0.88
	Control group	6.40	4.00	9.00	1.43	0.33	−0.09
Negative scale score	Intervention group	11.10	9.00	13.00	1.37	0.58	1.03
	Control group	8.90	7.00	11.00	1.04	0.55	−0.22
General autism scale score	Intervention group	27.90	26.00	30.00	1.14	0.08	−0.55
	Control group	21.60	19.00	23.00	1.28	0.33	−0.23
Unresponsive syndrome	Intervention group	5.30	4.00	7.00	0.90	0.22	−0.22
	Control group	4.30	3.00	5.00	0.64	0.19	−0.09
Thought disorder symptoms	Intervention group	5.80	4.00	8.00	1.08	0.66	−0.33
	Control group	4.50	3.00	6.00	0.81	0.22	−0.77
Active symptom cluster	Intervention group	5.40	4.00	7.00	0.80	1.11	1.24
	Control group	1.80	1.00	3.00	0.75	1.12	1.01
Depressive symptom group	Intervention group	7.10	3.00	9.00	0.94	0.28	−0.75
	Control group	7.20	6.00	9.00	0.98	0.55	−0.33
Total score	Intervention group	54.80	53.00	56.00	0.98	0.11	−1.08
	Control group	33.20	31.00	36.00	1.40	0.46	0.23

**Table 4:** Results of the post-psychiatric evaluation of the intervention and control groups.

Gross rating	Groups	Mean	Min	Max	Std	Skewness	Kurtosis
GAS score	Intervention group	90.80	87.00	94.00	2.27	0.22	−0.22
	Control group	88.70	87.00	91.00	1.19	0.19	−0.78
Positive and negative symptom scale							
Positive scale score	Intervention group	5.58	4.00	6.90	0.85	0.73	−0.69
	Control group	4.49	3.30	5.30	0.65	0.66	0.22
Negative scale score	Intervention group	5.57	4.00	7.00	0.86	0.33	−1.02
	Control group	4.47	3.30	5.30	0.62	−0.02	−0.99
General autism scale score	Intervention group	12.84	11.90	13.50	0.50	0.01	−1.12
	Control group	11.93	11.20	12.80	0.54	0.66	−0.09
Unresponsive syndrome	Intervention group	1.98	1.60	2.50	0.25	0.66	0.44
	Control group	1.86	1.60	1.99	0.13	−0.03	−0.88
Thought disorder symptoms	Intervention group	2.02	1.60	2.50	0.23	1.12	0.74
	Control group	1.87	1.60	2.02	0.14	0.38	−0.86
Active symptom cluster	Intervention group	1.02	0.88	1.20	0.09	1.14	0.34
	Control group	0.86	0.75	0.99	0.15	1.22	0.57
Depressive symptom group	Intervention group	3.04	2.70	3.50	0.22	0.77	0.37
	Control group	2.92	2.69	3.05	0.11	0.89	−0.01
Total score	Intervention group	23.01	22.60	23.50	0.25	0.02	−1.04
	Control group	22.45	22.10	23.01	0.29	0.23	−0.02

Moreover, two specialized nonverbal diagnostic tools, the Self-Portrait Drawing (SPD) and the Drawing Development Scale (DDS) were employed to capture the expressive features and symbolic content of participants’ creative outputs, serving as indicators of their underlying psychological and emotional states. Quantitative and visual analysis using SPSS 27.0 and Python revealed that the intervention group experienced a statistically significant enhancement in drawing performance (Tables 5–8). Their artworks displayed increasingly coherent structure, thematic richness, and emotional clarity. For instance, in the case of subject B, the DDS score in the final evaluation phase was reduced to below 0.40 (*M* = 0.06), a threshold indicating a transition toward neurotypical drawing patterns. Subject A’s creative progress—from scattered geometric figures to named narrative scenes such as “My Home”—not only reflected improvements in cognitive integration but also the emergence of a sense of identity and belonging.

**Table 5:** Independent samples *t*-test of pre- and post-test SPD scores between the intervention and control groups.

Subjects	Group (mean ± SD)		*t*	*p*
	Control group (*n* = 30)	Intervention group (*n* = 30)		
Pre-test SPD	33.03 ± 3.41	32.93 ± 3.69	0.109	0.914
Post-test SPD	34.13 ± 5.49	41.77 ± 6.85	−4.763	0.000**

*p < 0.05, **p < 0.01.

**Table 6:** Paired samples *t*-test of pre- and post-test SPD scores within the intervention group.

Paired (mean ± SD)		SD	*t*	*p*
Post-test SPD	Pre-test SPD			
41.77 ± 6.85	32.93 ± 3.69	8.83	7.387	0.000**

*p < 0.05, **p < 0.01.

**Table 7:** Independent samples *t*-test of pre- and post-test DDS scores between the intervention and control groups.

Subjects	Group (mean ± SD)		*t*	*p*
	Control group (*n* = 30)	Intervention group (*n* = 30)		
Pre-test DDS	68.50 ± 9.28	67.67 ± 5.02	0.433	0.667
Post-test DDS	68.67 ± 9.76	75.30 ± 6.88	−3.041	0.004**

*p < 0.05, **p < 0.01.

**Table 8:** Paired samples *t*-test of pre- and post-test DDS Scores within the intervention group.

Paired (mean ± SD)		SD	*t*	*p*
Post-test DDS	Pre-test DDS			
75.30 ± 6.88	67.67 ± 5.02	7.63	10.419	0.000**

*p < 0.05, **p < 0.01.

Following these findings, we evaluated participants’ self-perception and overall quality of life. The analysis of the pre-test and post-test results (Tables 9–12) showed that there were no significant differences between the two groups in terms of self-concept and psychological state. Among them, the symptom severity of the subjects in the EC-DT model group was significantly reduced after the intervention (GQOL-74 and TSCS), and the results had high internal consistency, indicating that the painting intervention based on embodied cognition can help significantly improve the psychological function of autistic students. The self-concept scores (TSCS) increased markedly (*Δ* = 29.37, *p* < 0.001), social skill functioning as measured by the Goal Attainment Scale (GAS) improved (Δ = 15.6, *p* = 0.003), and overall quality of life (GQOL-74) showed a substantial enhancement (Δ = 21.3, *p* < 0.001). These results provide empirical support for the effectiveness of embodied cognition-based drawing interventions in enhancing emotional, cognitive, and interpersonal functioning among autistic students.

**Table 9:** Independent samples t-test of pre- and post-intervention GQOL-74 scores between the groups.

Subjects	Group (mean ± SD)		*t*	*p*
	Control group (*n* = 30)	Intervention group (*n* = 30)		
Pre-test—sleep and energy	16.27 ± 5.47	15.90 ± 4.50	0.284	0.778
Pre-test—physical discomfort	13.13 ± 4.24	13.33 ± 3.36	−0.203	0.84
Pre-test—eating function	9.33 ± 3.69	9.03 ± 2.41	0.373	0.711
Pre-test—motor and sensory function	15.70 ± 5.50	16.37 ± 4.16	−0.529	0.599
Pre-test—mental tension	12.97 ± 3.84	12.50 ± 3.63	0.484	0.63
Pre-test—negative affect	12.23 ± 3.87	13.10 ± 3.69	−0.887	0.379
Pre-test—positive affect	9.07 ± 3.20	9.93 ± 3.02	−1.079	0.285
Pre-test—cognitive function	15.93 ± 4.76	17.17 ± 4.86	−0.992	0.325
Pre-test—self-esteem	11.87 ± 3.73	13.50 ± 3.66	−1.711	0.092
Pre-test—social support	12.63 ± 3.74	12.90 ± 3.58	−0.282	0.779
Pre-test—interpersonal skills	9.27 ± 2.98	9.40 ± 3.09	−0.17	0.866
Pre-test—work and study	9.07 ± 3.20	9.93 ± 3.02	−1.079	0.285
Pre-test—leisure and recreational life	9.33 ± 3.03	8.93 ± 3.08	0.507	0.614
Pre-test—marriage and family	14.97 ± 4.73	16.07 ± 4.83	−0.891	0.377
Post-test—sleep and energy	15.93 ± 5.36	17.50 ± 3.91	−1.294	0.201
Post-test—physical discomfort	13.10 ± 4.33	15.03 ± 2.55	−2.108	0.040*
Post-test—eating function	9.30 ± 3.66	10.00 ± 2.23	−0.895	0.375
Post-test—motor and sensory function	15.63 ± 5.49	17.53 ± 3.61	−1.584	0.12
Post-test—mental tension	12.77 ± 3.80	14.10 ± 2.81	−1.545	0.128
Post-test—negative affect	11.97 ± 3.85	14.23 ± 2.98	−2.552	0.013*
Post-test—positive affect	9.17 ± 3.26	10.87 ± 2.54	−2.252	0.028*
Post-test—cognitive function	16.00 ± 4.75	19.10 ± 3.42	−2.902	0.005**
Post-test—self-esteem	11.77 ± 3.78	14.60 ± 2.95	−3.232	0.002**
Post-test—social support	12.67 ± 3.64	14.17 ± 2.91	−1.762	0.083
Post-test—interpersonal skills	9.30 ± 2.98	10.83 ± 2.39	−2.195	0.032*
Post-test—work and study	9.17 ± 3.26	10.87 ± 2.54	−2.252	0.028*
Post-test—leisure and recreational life	9.43 ± 3.02	10.30 ± 2.79	−1.153	0.254
Post-test—marriage and family	15.00 ± 4.88	17.67 ± 4.16	−2.276	0.027*

*p < 0.05, **p < 0.01.

**Table 10:** Paired samples *t*-test of pre- and post-intervention GQOL-74 scores within the intervention group.

Subjects	Paired (mean ± SD)		SD	*t*	*p*
	Post-test	Pre-test			
Sleep and energy	17.50 ± 3.91	15.90 ± 4.50	1.6	2.174	0.038*
Physical discomfort	15.03 ± 2.55	13.33 ± 3.36	1.7	2.837	0.008**
Eating function	10.00 ± 2.23	9.03 ± 2.41	0.97	2.436	0.021*
Motor and sensory function	17.53 ± 3.61	16.37 ± 4.16	1.17	1.545	0.133
Mental tension	14.10 ± 2.81	12.50 ± 3.63	1.6	2.463	0.020*
Negative affect	14.23 ± 2.98	13.10 ± 3.69	1.13	2.416	0.022*
Positive affect	10.87 ± 2.54	9.93 ± 3.02	0.93	2.494	0.019*
Cognitive function	19.10 ± 3.42	17.17 ± 4.86	1.93	2.571	0.016*
Self-esteem	14.60 ± 2.95	13.50 ± 3.66	1.1	2.204	0.036*
Social support	14.17 ± 2.91	12.90 ± 3.58	1.27	2.109	0.044*
Interpersonal skills	10.83 ± 2.39	9.40 ± 3.09	1.43	2.46	0.020*
Work and study	10.87 ± 2.54	9.93 ± 3.02	0.93	2.494	0.019*
Leisure and recreational life	10.30 ± 2.79	8.93 ± 3.08	1.37	2.355	0.025*
Marriage and family	17.67 ± 4.16	16.07 ± 4.83	1.6	2.025	0.052

*p < 0.05, **p < 0.01.

**Table 11:** Independent samples *t*-test of pre- and post-test TSCS scores between the intervention and control groups after the art intervention.

Subjects	Group (mean ± SD)		*t*	*p*
	Control group (*n* = 30)	Intervention group (*n* = 30)		
Pre-test—physical self	50.47 ± 9.33	47.47 ± 11.44	1.113	0.27
Pre-test—moral-ethical self	50.00 ± 8.75	47.43 ± 11.31	0.983	0.33
Pre-test—psychological	50.63 ± 9.54	46.93 ± 11.18	1.379	0.173
Pre-test—family self	50.97 ± 9.75	47.80 ± 12.16	1.113	0.27
Pre-test—social self	50.07 ± 9.26	47.27 ± 11.46	1.041	0.302
Pre-test—self-criticism	41.50 ± 6.04	39.41 ± 8.02	1.131	0.263
Pre-test—identity	100.70 ± 18.97	94.40 ± 22.09	1.185	0.241
Pre-test—self-satisfaction	101.30 ± 17.12	94.70 ± 23.05	1.259	0.213
Pre-test—self-behavior	92.33 ± 17.08	87.40 ± 20.49	1.013	0.315
Pre-test—Grs (total score)	294.33 ± 52.76	276.50 ± 65.25	1.164	0.249
Post-test—physical self	47.37 ± 12.48	52.30 ± 7.08	−1.884	0.066
Post-test—moral-ethical	46.40 ± 12.10	52.97 ± 5.29	−2.724	0.010**
Post-test—psychological	46.53 ± 11.92	52.40 ± 6.00	−2.407	0.020*
Post-test—family self	46.77 ± 12.25	52.57 ± 6.21	−2.313	0.026*
Post-test—social self	46.37 ± 12.93	52.20 ± 6.02	−2.24	0.031*
Post-test—self-criticism	38.33 ± 8.51	44.17 ± 3.67	−3.447	0.001**
Post-test—identity	94.30 ± 24.79	104.57 ± 11.44	−2.06	0.046*
Post-test—self-satisfaction	93.27 ± 23.67	105.80 ± 11.23	−2.62	0.012*
Post-test—self-behavior	85.17 ± 21.95	95.50 ± 12.14	−2.257	0.029*
Post-test—Grs (total score)	272.73 ± 70.06	305.87 ± 34.33	−2.326	0.025*

*p < 0.05, **p < 0.01.

**Table 12:** Paired samples *t*-test of post-test TSCS scores within the intervention group after the art intervention.

Subjects	Paired (mean ± SD)		SD	*t*	*p*
	Post-test	Post-test			
Physical self	52.30 ± 7.08	47.47 ± 11.44	4.83	2.342	0.026*
Moral-ethical self	52.97 ± 5.29	47.43 ± 11.31	5.53	2.841	0.008**
Psychological	52.40 ± 6.00	46.93 ± 11.18	5.47	2.963	0.006**
Family self	52.57 ± 6.21	47.80 ± 12.16	4.77	2.093	0.045*
Social self	52.20 ± 6.02	47.27 ± 11.46	4.93	2.327	0.027*
Self-criticism	44.03 ± 3.66	39.41 ± 8.02	4.62	3.357	0.002**
Identity	104.57 ± 11.44	94.40 ± 22.09	10.17	2.524	0.017*
Self-satisfaction	105.80 ± 11.23	94.70 ± 23.05	11.1	2.818	0.009**
Self-behavior	95.50 ± 12.14	87.40 ± 20.49	8.1	2.272	0.031*
Grs (total score)	305.87 ± 34.33	276.50 ± 65.25	29.37	2.573	0.015*

*p < 0.05, **p < 0.01.

Complementing the quantitative findings, qualitative analyses of students’ verbal expressions and artwork revealed three salient themes: (1) embodied emotion regulation, where participants described experiences such as “feeling emotions flowing through fingertips when drawing circles,” indicating a somatosensory pathway of emotional expression; (2) cultural symbol–mediated communication, with children using colors like red to metaphorically express concepts such as “warmth” and “security,” highlighting the cultural and emotional resonance embedded in visual choices; and (3) enhanced self-representation in painting, reflecting a growing ability to construct and externalize identity through visual narratives. Case evidence further substantiated these themes. For instance, Subject A, a student with moderate autism, progressed from simple geometric shapes to complete scene depictions and, by the fifth week, spontaneously titled his work My Home. Subject B, with mild autism, demonstrated reduced line disorder and extended his group interaction from 3 min at baseline to 12 min; parents also reported that he began to actively use drawings to describe school experiences. Together, these cases illustrate how the EC-DT model not only mitigates core autism-related symptoms but also fosters multidimensional growth in cognition, emotion, and socialization.

The mechanism underlying these improvements can be understood through a “body–media–emotion” pathway. At the outset, participants externalized inner tension and feelings of entrapment through dense, chaotic lines. With continued engagement in structured drawing tasks, bodily actions such as repetitive line-making and intentional color selection mediated emotional regulation, gradually transforming confusion into order. The art medium thus became a vehicle for embodied expression, enabling participants to experience relief, heightened self-awareness, and restored emotional balance. This synergistic interplay of bodily motion, symbolic drawing, and culturally embedded metaphors provides a compelling rationale for including embodied, culturally responsive art therapy as a core component of autism intervention frameworks.

## 4 Discussion

### 4.1 Reconstruction mechanism of self-concept

Through embodied painting practices, autistic students demonstrated a more coherent and integrated self-representation, as evidenced by the significant improvement in SPD scores (*p* < 0.001). In particular, Subject A provided a concrete illustration of the mechanism described by Abrahamson (2014), namely that bodily movement facilitates cognitive integration. This was exemplified by his repeated adjustments to line intensity when painting the “My Home” scene, which reflects a real-time coupling between sensorimotor activity and visual feedback (Thelen and Smith, 1994). Such coupling may activate the mirror neuron system (Rizzolatti and Craighero, 2004), thereby enabling the transformation of a fragmented body schema into a more unified and embodied self-image. This developmental trajectory was clearly observable in Subject A’s self-portraits: from blurred facial features in the early stages (Figure 4A-02) to more detailed and complete facial representation later in the intervention (Figure 4A-06). The corresponding increase in SPD score by 41% (*p* < 0.001) aligns with the dynamic systems theory proposed by van Geert (1998), which posits that perception–action cycles are fundamental to cognitive reorganization.

Cultural specificity also played a crucial role in the reconstruction of self-concept. For instance, Subject B adopted the motif of a “sawtooth wave” to symbolize emotional release (Figure 6B-18), contrasting with Western studies that tend to rely on abstract visual elements (e.g., straight lines representing “stability”) as emotional metaphors (Hu et al., 2021). This contrast underscores the culturally embedded nature of visual symbolism. While the general findings resonate with global embodied cognition literature, they also highlight culturally grounded distinctions. For example, Chinese students were more inclined to use red to express “warmth” rather than “danger” (Chen and Chi, 2022), and to incorporate traditional motifs—such as circular patterns representing “harmony”—to express belongingness (Cui and Wang, 2022). These findings are consistent with the “embodied cultural symbol” theory proposed by Cui and Wang (2022). Parent interviews further validated this symbolic function; one parent noted that the child began to point to the painting and say, “This is me,” exemplifying how visual symbols serve as mediums of self-recognition for autistic individuals, a phenomenon that echoes the interpretation of art as a compensatory channel for identity construction (Fasulo, 2019).

Additionally, the cross-modal integration of tactile stimulation and visual representation proved to be particularly impactful. Subject B demonstrated significantly greater narrative coherence (*p* < 0.05) after converting tactile experiences (e.g., clay modeling) into visual symbols, supporting Dijkstra et al. (2014) theory of embodied metaphor, and likely implicating multisensory integration processes in the angular gyrus (Shams and Seitz, 2008). However, individuals with lower baseline social communication skills showed relatively limited gains, potentially due to weaker connectivity within the default mode network (Uddin et al., 2013). This suggests a need for future personalized interventions that integrate neurofeedback techniques to optimize embodiment-based therapies (Kırca, 2019).

### 4.2 Theoretical expansion and localized innovation

This study provides a new paradigm for the localization of art therapy. Subject B’s self-criticism (SC) score decreased (*p* = 0.002) and line disorder decreased by 58% (*p* < 0.001), indicating that the “body–medium–emotion” connection mechanism can break through the limitations of the Western DDS model (Baron-Cohen et al., 1985). For example, the use of “reciprocating patterns” in his paintings (Figure 6B-12) not only enhances the effectiveness of emotional expression but also aligns with the Chinese cultural ideal of “harmony between man and nature” (Sgorbati, 2025). Unlike the more analytical, symbol-heavy logic often seen in Western approaches, this body-based, culturally resonant method draws on lived experience and embedded meaning. It suggests that cognitive reorganization does not always need to be top-down—sometimes, it flows through the brush.

It’s a clash of values: collectivism versus individualism, closeness versus autonomy. Such findings underscore the necessity of culturally adaptive interventions. Future research could dig deeper into urban–rural differences in China, for instance, how access to traditional art training varies, or look at cross-cultural contrasts in symbolic systems between East and West. To push boundaries further, dynamic environments combining traditional symbols with VR technology, like simulating the blurred realism of Chinese ink painting, should be developed to evoke stronger cultural resonance and sensory immersion.

### 4.3 Limitations and prospects

Although the model demonstrated short-term effects, individual reactions such as vomiting and hallucinations (Subject B) suggest that intervention intensity requires careful calibration. These responses should not be interpreted as failures but as signals for adjustment. Moreover, the unique visual languages of autistic students—their colors, symbols, and line rhythms—should not be dismissed as mere symptoms; rather, they represent alternative cognitive pathways (Martínez-Vérez et al., 2024). Despite these insights, several limitations must be acknowledged. The sample size was modest (*n* = 60). Although prior literature indicates that comparable sample sizes can yield meaningful results, the restricted number of participants, combined with WISC-IV scores ranging from 50 to 85, limits the generalizability of the findings to populations with broader intellectual variation (Kilroy et al., 2021). Recruitment challenges, strict inclusion criteria, and resource constraints further restricted statistical inference. In addition, while participants reported minimal exposure to concurrent therapies, the limited exploration of potential confounders—such as family dynamics, parental involvement, and out-of-school treatments (e.g., speech therapy, behavioral training)—reduces the study’s transparency and may have influenced the observed outcomes (Strang, 2024).

Future research should address these limitations by expanding sample size, including more heterogeneous participants, and conducting longitudinal follow-ups (e.g., 6–12 months) to determine the sustainability of improvements. More rigorous monitoring of concurrent interventions and family contexts is needed to disentangle the specific effects of art-based therapy. Beyond traditional ink painting, structured training paradigms incorporating other art forms (e.g., printmaking, lacquer painting) and tools may reveal differentiated therapeutic mechanisms. Additionally, combining behavioral outcomes with neuroimaging approaches such as fMRI will further validate the neural mechanisms of embodied cognition and emotion regulation (Kilroy et al., 2021). Collectively, these directions highlight the promise of culturally grounded, embodied art therapy as both a scientifically robust and contextually adaptive model for autism intervention.

## 5 Conclusion

Grounded in embodied cognition theory EC-DT model suggests that autistic students can significantly enhance self-concept, social functioning, and overall quality of life through the perception–action loop and embodied cultural expression. Synergistic coordination between hand movement and visual feedback was shown to reorganize fragmented cognition, while culturally rooted symbols, such as circular patterns and red as a metaphor for warmth, amplified the emotional resonance of non-verbal communication.

Based on these findings, we outline three translational directions: (1) develop embodied assessment tools that convert visual characteristics (e.g., line texture, color diffusion) into emotional regulation biomarkers; (2) establish culturally responsive intervention frameworks leveraging traditional symbolic systems; and (3) design immersive therapeutic spaces using adaptive sensory stimuli to reinforce sensorimotor coupling. These strategies build on sensory integration theory while offering a localized alternative to prevailing Western-centric models.

Limitations include the single-institution sample, limited follow-up duration (3 months), and absence of neurophysiological measurements. Future work should pursue longitudinal, multi-site studies and incorporate VR-based simulations of traditional aesthetics to investigate the neural mechanisms underlying culturally embedded therapeutic processes.

## Data Availability

The raw data supporting the conclusions of this article will be made available by the authors, without undue reservation.
